# Diagnostic Performance of Complete Blood Count Derived Indices for Acute Coronary Syndrome in Emergency Department Patients With Acute Chest Pain: A Single‐Center Retrospective Diagnostic Accuracy Study

**DOI:** 10.1002/hsr2.73333

**Published:** 2026-10-05

**Authors:** Mohammad Ghorbani, Mostafa Shakeri, Elahe Rahimi, Ebrahim Salavati, Raheleh Derafshi, Mohamad Esmail Gheydari, Amirhossein Gholizadeh Behabadi

**Affiliations:** ^1^ Department of Medical Laboratory Sciences, Faculty of Medicine Gonabad University of Medical Sciences Gonabad Iran; ^2^ Faculty of Medicine Gonabad University of Medical Sciences Gonabad Iran; ^3^ Allameh Bohlool Hospital Gonabad University of Medical Sciences Gonabad Iran; ^4^ Department of Pediatric Gastroenterology, Faculty of Medicine Gonabad University of Medical Sciences Gonabad Iran; ^5^ Department of Cardiology, Taleqani Hospital Shahid Beheshti University of Medical Sciences Tehran Iran; ^6^ Nutrition Department, Faculty of Medicine Gonabad University of Medical Sciences Gonabad Iran

**Keywords:** acute coronary syndrome, diagnostic value, hematologic indices

## Abstract

**Background and Aims:**

Acute coronary syndrome (ACS), as one of the cardiovascular diseases, plays a significant role in patient mortality. Although appropriate diagnostic methods have been introduced for ACS, it still remains a challenge for physicians. Therefore, utilizing additional markers and ratios for the diagnosis of ACS can be beneficial. Hence, our study was conducted with the aim of evaluating the diagnostic value of indices and ratios derived from the complete blood count (CBC) test.

**Methods:**

In this retrospective diagnostic accuracy study, 360 patients presenting with acute chest pain to the emergency department of Allameh Bohlool Hospital were evaluated. Patients were classified into ACS (*n* = 167) and Non‐ACS (*n* = 193) groups based on the final clinical diagnosis. CBC‐derived parameters, including leukocyte count, neutrophil count, monocyte count, platelet count, mean platelet volume (MPV), neutrophil‐to‐lymphocyte ratio (NLR), monocyte‐to‐lymphocyte ratio (MLR), and platelet‐to‐lymphocyte ratio (PLR), were assessed. Diagnostic performance was evaluated using receiver operating characteristic (ROC) curve analysis. Multivariable logistic regression models were developed to determine independent associations and assess the incremental diagnostic value of CBC‐derived markers beyond clinical variables.

**Results:**

Patients with ACS had significantly higher leukocyte count, neutrophil count, monocyte count, MPV, NLR, and MLR levels compared with Non‐ACS patients (all *p* < 0.01). Among individual biomarkers, neutrophil count demonstrated the highest diagnostic performance (AUC = 0.720; 95% CI, 0.668–0.772), followed by leukocyte count (AUC = 0.704; 95% CI, 0.651–0.758) and NLR (AUC = 0.669; 95% CI, 0.613–0.725). After adjustment for clinical variables, leukocyte count (adjusted OR = 1.64; 95% CI, 1.41–1.91; *p* < 0.001) and neutrophil count (adjusted OR = 1.68; 95% CI, 1.42–1.99; *p* < 0.001) remained independently associated with ACS. Addition of leukocyte count to the clinical model significantly improved discrimination (AUC increased from 0.787 to 0.850; ΔAUC = 0.063; DeLong *p* < 0.001).

**Conclusion:**

In this study, selected CBC‐derived parameters, particularly neutrophil count, leukocyte count, and NLR, showed statistically significant but limited discriminatory ability for ACS. These indices may be considered adjunctive inflammatory markers, but their diagnostic performance is insufficient for use as stand‐alone tests and should be interpreted alongside clinical assessment, ECG, and cardiac troponin testing.

## 1 Introduction

Cardiovascular disease (CVD) remains one of the leading causes of mortality worldwide [1, 2, 3, 4]. In In the United States, more than one million individuals develop acute coronary syndrome (ACS) annually, and approximately 400,000 deaths each year are attributed to coronary artery disease. Similarly, the prevalence of CVD has increased substantially in our country, where CVD accounts for about 45% of deaths, 26% of years of life lost, and 10.4% of the total disease burden [5].

Currently, the diagnosis of acute myocardial infarction (AMI) is based on the presence of chest pain in conjunction with dynamic electrocardiographic (ECG) changes and elevated protein biomarkers. Among these, cardiac troponin I, myoglobin (Mb), and creatine kinase‐MB (CK‐MB) are the most frequently used indicators for the clinical diagnosis of AMI [4, 6]. Despite substantial progress in the diagnosis and management of ACS, identifying myocardial ischemia continues to present a clinical challenge. Many low‐risk patients with suspected myocardial ischemia are admitted unnecessarily. Troponin, along with ECG, is the main diagnostic criterion for ACS, but this indicator can be false‐positive in cases such as sepsis, rhabdomyolysis, shock, embolism, etc [7]. The lack of laboratory kits, the high cost of the kit in some countries, and the skill of the laboratory expert can also make troponin measurement challenging. Challenges such as specificity, standardization, and interpretation of results also remain [8]. In a number of cases of ACS, no changes are observed in the ECG or non‐specific changes are observed, which can make the diagnosis of ACS challenging. As a result, the presence of a low‐cost adjunctive marker can help to evaluate diagnostic discrimination, and diagnostic protocols incorporating multiple biomarkers may improve the accuracy of ACS detection [9].

ACS encompasses a broad clinical spectrum, ranging from unstable angina (UA) to myocardial infarction with or without ST‐segment elevation [9].

AMI typically results from the rupture of an unstable atherosclerotic plaque within the coronary arteries. Such plaques often undergo local rupture or erosion, leading to platelet aggregation, thrombus formation, and coronary occlusion [6, 9, 10, 11]. The pathogenesis of atherosclerosis involves three major stages: dysregulated lipid metabolism, aberrant blood cell responses, and chronic inflammation of the vascular wall [12, 13]. Intercellular communication among various cellular mediators plays a crucial role in the initiation and progression of atherosclerotic plaque formation [14, 15]. Inflammation is a central mechanism in the development of atherosclerosis, the onset of ACS, and other CVD. Inflammatory markers such as leukocyte count, particularly the neutrophil‐to‐lymphocyte ratio (NLR), are important predictors of cardiovascular events [11, 16, 17]. Platelets also play a vital role in the pathogenesis of atherosclerosis and thrombus formation following plaque rupture. Platelet activation releases free arachidonic acid, which can be converted into prostaglandins such as thromboxane A_2_—a potent vasoconstrictor and platelet‐aggregating agent—or into leukotrienes, which further amplify the acute inflammatory response [18, 19].

The leukocyte count serves as a cellular marker of systemic inflammation [20]. The The NLR has been investigated across numerous clinical contexts as an indicator of chronic systemic inflammation [21]. Mean platelet volume (MPV), an index reflecting platelet size and activity, has been shown to increase in patients with conditions within the spectrum of ACS [22].

Muhammad Saleem Awan et al. utilized the NLR to predict troponin I elevation in patients with suspected non‐ST‐segment elevation ACS [16]. In another study, Randhir Pal et al. assessed MPV as a supportive diagnostic predictor in ACS patients [18]. Identifying additional markers that enable clinicians to anticipate rises in cardiac biomarkers could therefore be valuable. The complete blood count (CBC) is a simple, accessible, and inexpensive test that may have potential utility for this purpose [23, 24].

Accordingly, considering the above evidence and the widespread availability of the CBC test—which can be performed in any standard laboratory—this study aims to investigate the relationship between hematological markers and ratios, and the occurrence and diagnosis of AMI. Contemporary emergency department (ED) assessment of suspected ACS relies on clinical evaluation, ECG, and serial high‐sensitivity cardiac troponin algorithms; therefore, any CBC‐derived index should be interpreted as a potential adjunct rather than a substitute for validated diagnostic pathways [25]. Furthermore, this study extends previous research by examining a broader range of hematological indices and providing a more comprehensive assessment of their diagnostic potential in ACS. Ultimately, we aim to determine the extent to which hematological indices possess diagnostic value in identifying ACS and to evaluate their potential use as an adjunct index in aiding the early suspicion of this condition.

## 2 Materials and Methods

### 2.1 Study Patients

This single‐center retrospective diagnostic accuracy study was conducted from March 20, 2024, to March 6, 2025, in the ED and cardiac care unit (CCU) of Allameh Bohlool Hospital in Gonabad, Iran. A total of 360 patients (357 adults and 3 adolescents) were enrolled in the study after providing informed consent to participate. For adolescent participants, written informed consent was obtained from their parents or legal guardians, and written assent was obtained from the participants prior to their enrollment in the study. ECG was obtained within the first 30 min of admission using a DENA650 device (and a YASHAM635 device in the CCU). In high‐risk cases, repeat ECGs were performed at 20‐min intervals. Serum troponin levels were measured twice, with the first sample obtained at admission and the second collected 3–6 h later. According to Figure 1, patients presenting to the ED with acute chest pain who had available ECG and troponin test results and provided written informed consent were included in the study. Exclusion criteria comprised: 1. pericarditis, pleurisy, and myocarditis; 2. chronic kidney or liver disease; 3. hematologic disorders or malignancy; 4. multiple sclerosis (MS), rheumatologic disorders, inflammatory bowel disease (IBD), and any other inflammatory diseases; 5. platelet disorders; 6. active infection; 7. use of medications affecting CBC parameters (systemic long‐term corticosteroids, etc.); 8. missing CBC results; 9. incomplete medical records. Ethical approval for this study was obtained from the Ethics Committee of the Research Deputy of Gonabad University of Medical Sciences. Diagnosis of ACS was established according to the fourth universal definition of myocardial infarction (UDMI) and current European Society of Cardiology (ESC) or American Heart Association (AHA)/American College of Cardiology (ACC) guidance, using clinical presentation, serial ECG, serial cardiac troponin, and cardiologist assessment (Reference Standard); based on the above, patients were divided into STEMI, NSTEMI, and unstable angina, which were then classified into one group called patients with ACS. Patients with other conditions such as valvular heart disease, costochondritis, musculoskeletal pain, digestive system pains such as reflux, palpitation, psychological pains, pulmonary embolism, and non‐inflammatory diseases that cause chest pain were categorized as the Non‐ACS group. All patients were assigned a unique medical record and serial number, and relevant data were retrieved from the Hospital Information System (HIS) of Allameh Bohlool Hospital, supported by Rahavard Rayaneh Company (registration no. 78676).

**Figure 1:**
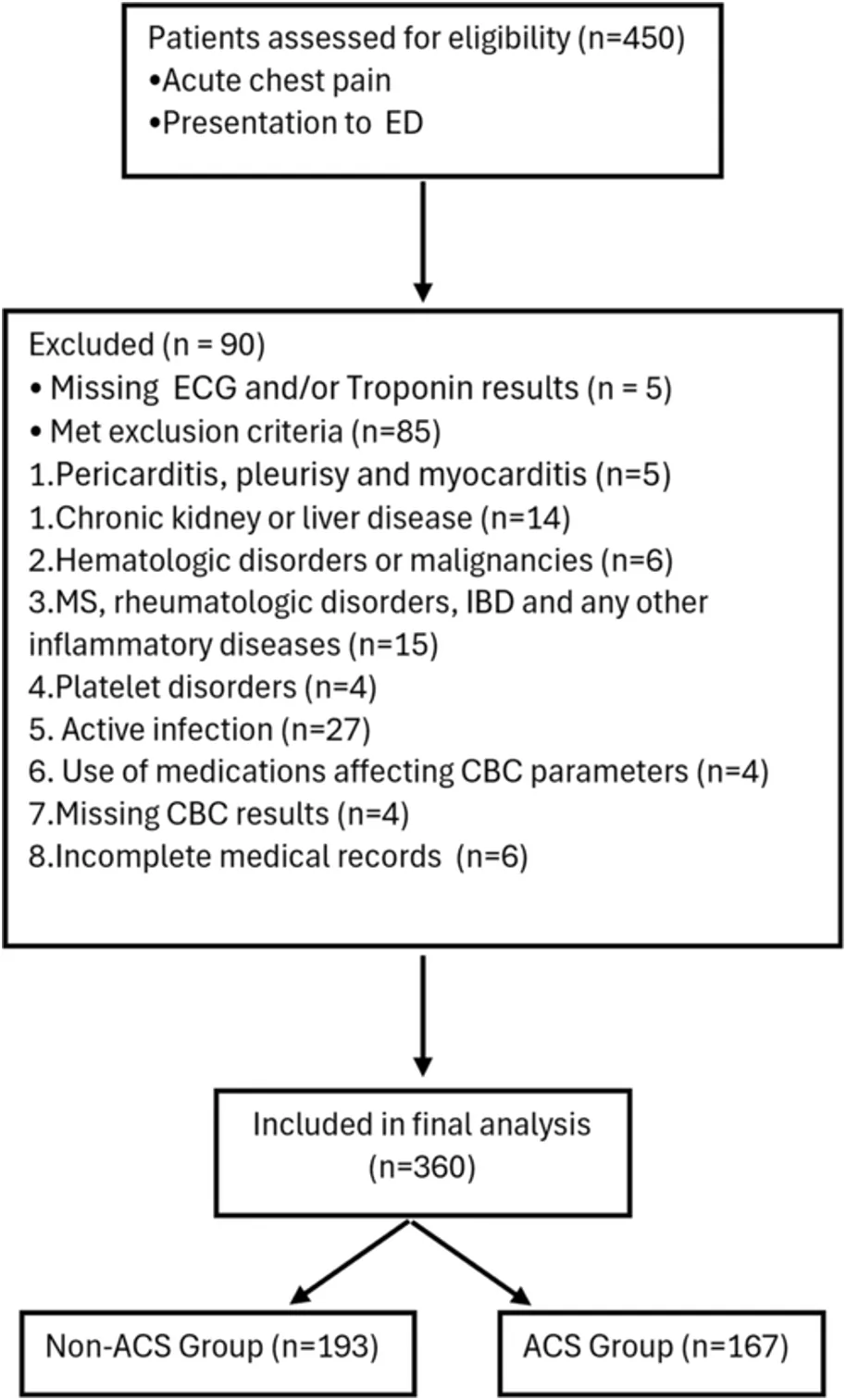
Flow diagram of patient selection. ACS was diagnosed according to UDMI and current ESC/AHA‐ACC guidelines.

### 2.2 Biochemical Evaluation

Approximately 5 mL of venous blood was drawn from each participant into tubes containing ethylenediaminetetraacetic acid (EDTA) and analyzed using standard instruments in the hospital's central laboratory. CBC samples, as the index test, were obtained at presentation before administration of therapeutic interventions. All CBC analyses were performed within 1 h of blood sample collection to minimize pre‐analytical variability. CBC analyses were performed using SYSMEX XP‐300, MINDRAY, and Cell TAK G analyzers. Quantitative troponin I measurements were conducted using Troponin I Chrome, RAMP, and VIDAS kits. Approximately 90% of the CBC and troponin tests were performed using the SYSMEX XP‐300 analyzer and VIDAS kits. On the occasions when these instruments or kits were unavailable, a small proportion of the tests were performed using the other analyzers and kits. Quality control of all analyzers was performed using control blood samples, Westgard rules, and Levey–Jennings charts.

Absolute leukocyte counts (including neutrophils, monocytes, and lymphocytes) and platelet indices (MPV and platelet count) were obtained. Subsequently, NLR, monocyte‐to‐lymphocyte ratio (MLR), and platelet‐to‐lymphocyte ratio (PLR) were calculated for further analysis.

### 2.3 Statistical Analysis

Statistical analyses were performed using IBM SPSS Statistics version 27.0 (IBM Corp., Armonk, NY, USA) and R version 4.5.0. Continuous variables were assessed for normality using the Shapiro–Wilk test and are presented as mean ± SD or median (IQR), as appropriate. Between‐group comparisons were performed using the independent‐samples t‐test or Mann–Whitney U test, while categorical variables were compared using the Pearson Chi‐square test. Diagnostic performance was evaluated using receiver operating characteristic (ROC) curve analysis. Area under the curve (AUC) with 95% confidence intervals, optimal cut‐off values (Youden index), sensitivity, specificity, positive predictive value, and negative predictive value were calculated. Multivariable logistic regression analysis was performed to identify independent predictors of ACS and to assess the incremental diagnostic value of each CBC‐derived marker after adjustment for clinical variables. Model discrimination was compared using DeLong's test, and calibration was assessed using the Hosmer–Lemeshow test. All tests were two‐sided, and a significance level of less than 0.05 was considered statistically significant.

## 3 Results

A total of 360 individuals (170 women and 190 men) were included in this study. The mean age of the participants was 58.63 ± 15.91 years, ranging from 13 to 99 years. The baseline characteristics of the study population are summarized in Table 1. The prevalence of history of diabetes was significantly higher in ACS patients (34.1% vs. 14.5%, *p* < 0.001). Similarly, history of hypertension was more frequent in ACS (49.7% vs. 33.2%, *p* = 0.002). History of family heart disease was also significantly associated with ACS (20.4% vs. 7.8%, *p* < 0.001). In addition, history of coronary stent implantation (16.2% vs. 8.3%, *p* = 0.03) and history of medication use (62.9% vs. 42.5%, *p* < 0.001) were more common among ACS patients. Other variables, including history of dyslipidemia, history of CVD, and history of coronary angiography, did not show statistically significant differences.

**Table 1:** Baseline characteristics of ACS group and Non‐ACS group.

Variable	Total	ACS *n* = 167	Non‐ACS *n* = 193	*p*‐value^a^
Age, years (mean ± SD)	58.63 ± 15.91	64.72 ± 12.21	53.35 ± 16.86	< 0.001
Female gender, *n* (%)	170 [47]	66 (39.5)	104 (53.9)	0.009
Smoker, *n* (%)	21 (5.0)	17 (10.0)	4 (2.1)	0.002
Diabetes, *n* (%)	85 (23.0)	57 (34.1)	28 (14.5)	< 0.001
Hypertension, *n* (%)	147 (40.0)	83 (49.7)	64 (33.2)	0.002
Dyslipidemia, *n* (%)	77 (21.0)	43 (25.7)	34 (17.6)	0.08
Family heart disease *n* (%)	49 (13.0)	34 (20.4)	15 (7.8)	< 0.001
Cardiovascular disease, *n* (%)	110 (30.0)	51 (30.5)	59 (30.6)	0.99
Coronary angiography, *n* (%)	98 (27.0)	45 (26.9)	53 (27.6)	0.98
Coronary stent, *n* (%)	43 (11.0)	27 (16.2)	16 (8.3)	0.03
Medication use, *n* (%)	187 (51.0)	105 (62.9)	82 (42.5)	< 0.001

Abbreviation: SD, standard deviation.

^a^ Independent sample t test was used for quantitative variables, and Pearson Chi‐square was used for qualitative variables.

The laboratory findings are summarized in Table 2. Patients with ACS were found to have higher median leukocyte counts (8.1 [6.5–9.7] ×10^3^/μL vs. 6.5 [5.3–7.7] ×10^3^/μL; *p* < 0.001), neutrophil counts (5 [3.90–6.60] ×10^3^/μL vs. 3.6 [2.93–4.90] ×10^3^/μL; *p* < 0.001), monocyte counts (0.4 [0.30–0.51] ×10^3^/μL vs. 0.31 [0.22–0.48] ×10^3^/μL; *p* = 0.004), MPV (10 [9.30–10.80] vs. 9.8 [9.10–10.20]; *p* = 0.003), NLR (2.64 [1.68–3.53] vs. 1.84 [1.32–2.56]; *p* < 0.001), and MLR (0.19 [0.13–0.29] vs. 0.17 [0.1–0.25]; *p* = 0.002) compared with the Non‐ACS group.

**Table 2:** Distribution of hematological parameters among ACS group and Non‐ACS group.

Variable	ACS median (IQR)	Non‐ACS median (IQR)	*p*‐value^a^
Leukocyte (×10^3^/μ L)	8.1 (6.5–9.7)	6.5 (5.3–7.7)	< 0.001
Neutrophil (×10^3^/μ L)	5.0 (3.90–6.60)	3.6 (2.93–4.90)	< 0.001
Lymphocyte (×10^3^/μ L)	2.0 (1.60–2.60)	2.1 (1.65–2.6)	0.41
Monocyte (×10^3^/μ L)	0.40 (0.30–0.51)	0.31 (0.22–0.48)	0.004
PLT (×10^3^/μ L)	227 (196–264)	225 (183–269)	0.94
MPV	10.00 (9.30–10.80)	9.80 (9.10–10.20)	0.003
NLR	2.64 (1.68–3.53)	1.84 (1.32–2.56)	< 0.001
MLR	0.19 (0.13–0.29)	0.17 (0.1–0.25)	0.002
PLR	108.66 (82.03–152.67)	107.83 (84.78–144.09)	0.75

Abbreviations: MLR, monocyte‐to‐lymphocyte ratio; MPV, mean platelet volume; NLR, neutrophil to lymphocyte ratio; PLR, platelet to lymphocyte ratio; PLT, platelet.

^a^ The Mann–Whitney U test was used for quantitative variables.

There were no significant differences in lymphocyte counts (2 [1.60–2.60] ×10^3^/μL vs. 2.1 [1.65–2.6] ×10^3^/μL; *p* = 0.41), platelet counts (227 [196–264] ×10^3^/μL vs. 225 [183–269] ×10^3^/μL; *p* = 0.94), or PLR (108.66 [82.03–152.67] vs. 107.83 [84.78–144.09]; *p* = 0.75) between the two groups.

ROC curve analysis (Table 3 and Figures 2 and Figures 3) demonstrated that neutrophil count showed the highest discriminatory ability among the evaluated CBC‐derived biomarkers for identifying ACS (AUC = 0.720, 95% CI: 0.668–0.772; *p* < 0.001), followed by leukocyte count (AUC = 0.704, 95% CI: 0.651–0.758; *p* < 0.001) and NLR (AUC = 0.669, 95% CI: 0.613–0.725; *p* < 0.001), indicating modest discriminatory ability. Monocyte count (AUC = 0.599, 95% CI: 0.540–0.658; *p* = 0.001), MPV (AUC = 0.591, 95% CI: 0.532–0.650; *p* = 0.003), and MLR (AUC = 0.593, 95% CI: 0.534–0.652; *p* = 0.002) demonstrated limited discriminatory ability. The optimal cut‐off values derived from ROC analysis were 3.79 × 10^3^/μL for neutrophils, 7.65 × 10^3^/μL for leukocyte, 2.47 for NLR, 0.40 × 10^3^/μL for monocytes, 10.75 for MPV, and 0.11 for MLR (Table 3). Detailed estimates of sensitivity, specificity, positive predictive value, and negative predictive value for each marker are presented in Table 3. Given the modest‐to‐limited discriminatory ability of the individual CBC‐derived biomarkers, their incremental discrimination beyond baseline demographic and cardiovascular risk factors was further assessed using multivariable logistic regression models.

**Table 3:** ROC analysis of main and ratio hematological markers in ACS patients.

Variable	AUC (95% CI)	Optimal cut‐off	SE of AUC	Sensitivity (%)	Specificity (%)	Positive‐PV (%)	Negative‐PV (%)	*p*‐value
Leukocycte	0.704 (0.651–0.758)	7.65	0.027	58.7	74.6	66.7	67.7	< 0.001
Neutrophil	0.720 (0.668–0.772)	3.79	0.027	79.0	53.4	55.4	74.6	< 0.001
Monocyte	0.599 (0.540–0.658)	0.40	0.030	58.2	60.6	55.8	63	0.001
MPV	0.591 (0.532–0.650)	10.75	0.030	26.9	89.1	68.2	58.5	0.003
NLR	0.669 (0.613–0.725)	2.47	0.029	55.7	74.6	65.5	66.1	< 0.001
MLR	0.593 (0.534–0.652)	0.11	0.030	85.5	32.6	52	72.4	0.002

Abbreviations: AUC, area under the curve; PV, predictive value; SE, standard error.

**Figure 2:**
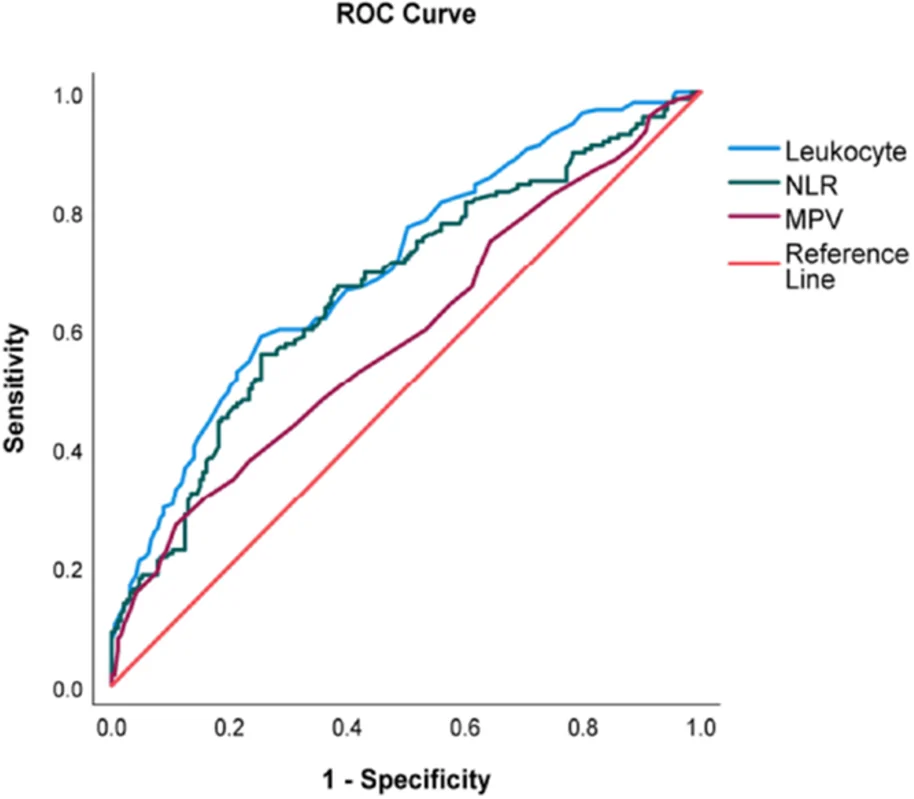
Receiver operating characteristic (ROC) curves showing the discriminatory ability of leukocyte count, neutrophil‐to‐lymphocyte ratio (NLR), and mean platelet volume (MPV) for distinguishing patients with ACS from Non‐ACS. The x‐axis represents 1 − specificity, and the y‐axis represents sensitivity. The diagonal reference line represents a non‐discriminatory test (AUC = 0.50). The discriminatory ability of each biomarker was evaluated using the area under the ROC curve (AUC), with larger AUC values indicating better discriminatory ability. The corresponding AUC, sensitivity, specificity, and optimal cutoff values for each biomarker are presented in Table 3.

**Figure 3:**
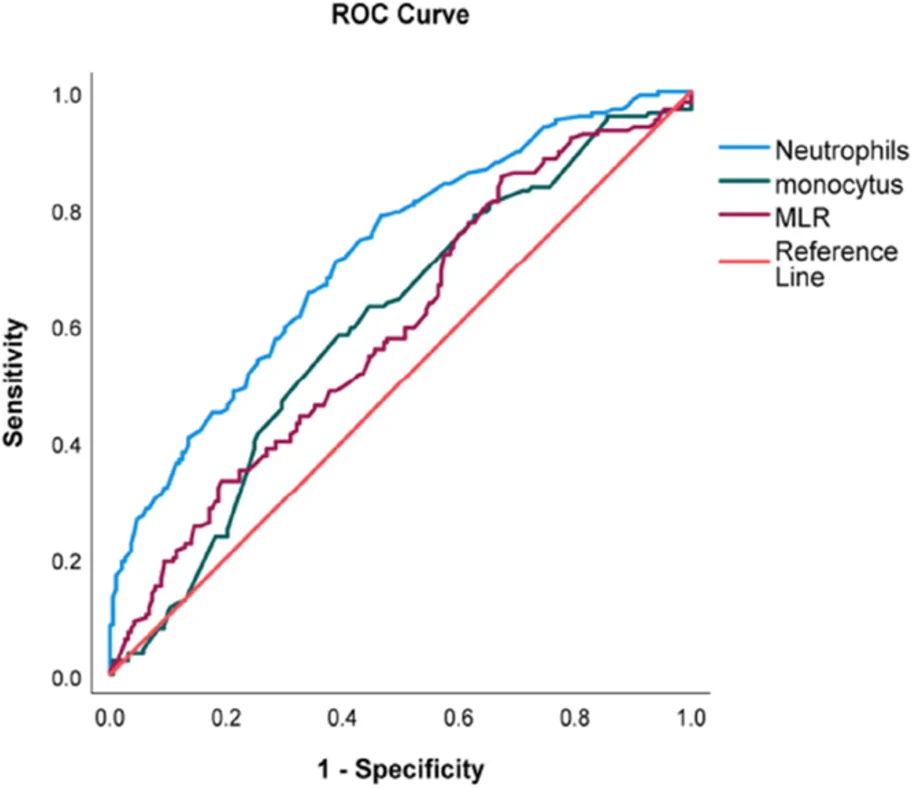
Receiver operating characteristic (ROC) curves showing the discriminatory ability of neutrophil count, monocyte count, and the monocyte‐to‐lymphocyte ratio (MLR) for distinguishing patients with ACS from Non‐ACS. The x‐axis represents 1 − specificity, and the y‐axis represents sensitivity. The diagonal reference line represents a non‐discriminatory test (AUC = 0.50). The discriminatory ability of each biomarker was evaluated using the area under the ROC curve (AUC), with larger AUC values indicating better discriminatory ability. The corresponding AUC, sensitivity, specificity, and optimal cutoff values for each biomarker are presented in Table 3.

To account for potential confounding factors, a multivariable logistic regression model including age, sex, smoking status, diabetes, hypertension, family history of heart disease, and previous coronary stent implantation was constructed (Table 4). This model was defined as the baseline model and included demographic and cardiovascular risk factors. After adjustment for these baseline factors, increasing age (adjusted OR 1.06, 95% CI 1.04–1.08; *p* < 0.001), male sex (adjusted OR 2.48, 95% CI 1.49–4.14; *p* < 0.001), smoking (adjusted OR 5.20, 95% CI 1.54–17.57; *p* = 0.008), diabetes mellitus (adjusted OR 2.50, 95% CI 1.41–4.44; *p* = 0.002), and a family history of heart disease (adjusted OR 2.87, 95% CI 1.40–5.92; *p* = 0.004) were independently associated with ACS. In contrast, hypertension (adjusted OR 1.27, 95% CI 0.74–2.17; *p* = 0.38) and previous coronary stent implantation (adjusted OR 1.31, 95% CI 0.62–2.75; *p* = 0.49) were not independently associated with ACS.

**Table 4:** Multivariable logistic regression analysis of baseline cardiovascular risk factors associated with ACS.

Variable	Adjusted OR	95% CI	*p*‐value
Age, years	1.06	1.04–1.08	< 0.001
Male gender	2.48	1.49–4.14	< 0.001
Smoker	5.20	1.54–17.57	0.008
Diabetes	2.50	1.41–4.44	0.002
Hypertension	1.27	0.74–2.17	0.38
Family heart disease	2.87	1.40–5.92	0.004
Coronary stent	1.31	0.62–2.75	0.49

Abbreviations: CI, confidence interval; OR, odds ratio.

To evaluate whether CBC‐derived hematological markers were independently associated with ACS beyond baseline cardiovascular risk factors, each marker was entered separately into a multivariable logistic regression model adjusted for age, sex, smoking status, diabetes, hypertension, family history of heart disease, and previous coronary stent implantation (Table 5). After adjustment, leukocyte count (per 1 × 10^3^/µL increase) (adjusted OR 1.64, 95% CI 1.41–1.91; *p* < 0.001), neutrophil count (per 1 × 10^3^/µL increase) (adjusted OR 1.68, 95% CI 1.42–1.99; *p* < 0.001), monocyte count (per 1 × 10^3^/µL increase) (adjusted OR 3.61, 95% CI 1.07–12.12; *p* = 0.038), MPV (per 1 fL increase) (adjusted OR 1.43, 95% CI 1.10–1.86; *p* = 0.008), and NLR (per 1‐unit increase) (adjusted OR 1.38, 95% CI 1.15–1.66; *p* = 0.001) remained independently associated with ACS. In contrast, MLR (per 1‐unit increase) was not independently associated with ACS after adjustment (adjusted OR 1.89, 95% CI 0.26–13.99; *p* = 0.54).

**Table 5:** Multivariable logistic regression analysis of CBC‐derived hematological markers adjusted for baseline cardiovascular risk factors.

CBC‐derived marker (unit of increase)	Adjusted OR	95% CI	*p*‐value
Leukocyte (per 1 × 10^3^/µL increase)	1.64	1.41–1.91	< 0.001
Neutrophil (per 1 × 10^3^/µL increase)	1.68	1.42–1.99	< 0.001
Monocyte (per 1 × 10^3^/µL increase)	3.61	1.07–12.12	0.04
MPV (per 1 fL increase)	1.43	1.10–1.86	0.008
NLR (per 1 fL increase)	1.38	1.15–1.66	0.001
MLR (per 1 fL increase)	1.89	0.26–13.99	0.54

To determine whether CBC‐derived hematological markers provide incremental diagnostic value beyond baseline cardiovascular risk factors, the discrimination of the multivariable models was compared using ROC curve analysis and DeLong's test (Table 6). The baseline model alone demonstrated an AUC of 0.787 (95% CI, 0.741–0.833). Addition of leukocyte count significantly improved the model's discriminative performance (AUC = 0.850, 95% CI, 0.811–0.888; ΔAUC = 0.063; DeLong *p* < 0.001), yielding the highest AUC and the highest Nagelkerke R^2^ (0.463) among all evaluated models, with no evidence of poor fit according to the Hosmer–Lemeshow test (*p *= 0.72). Similarly, incorporation of neutrophil count significantly increased the AUC to 0.842 (95% CI, 0.802–0.882; ΔAUC = 0.055; DeLong *p*< 0.001), although the Hosmer–Lemeshow test showed a borderline result (*p *= 0.06). In contrast, adding NLR, MPV, monocyte count, or MLR did not result in a statistically significant improvement in discrimination compared with the baseline model alone (all DeLong *p*> 0.05), despite modest increases in AUC for NLR, MPV, and monocyte count. Notably, the Baseline + MPV model showed evidence of lack of fit according to the Hosmer–Lemeshow test (*p *= 0.02), and its calibration should therefore be interpreted with caution. The ROC curves of the baseline model, the baseline + leukocyte model, and the baseline + neutrophil model are presented in Figure 4.

**Table 6:** Comparison of multivariable diagnostic models for predicting ACS.

Model	AUC (95% CI)	ΔAUC versus Clinical	DeLong *p*‐value	Nagelkerke R^2^	Hosmer–Lemeshow *p*‐value
Baseline model	0.787 (0.741–0.833)	Reference	—	0.320	0.74
Baseline +Leukocyte	0.850 (0.811–0.888)	+0.063	< 0.001	0.463	0.72
Baseline +Neutrophil	0.842 (0.802–0.882)	+0.055	< 0.001	0.447	0.06
Baseline + NLR	0.803 (0.758–0.847)	+0.016	0.07	0.358	0.74
Baseline + MPV	0.801 (0.756–0.846)	+0.014	0.07	0.341	0.02
Baseline + Monocyte	0.793 (0.747–0.839)	+0.006	0.28	0.332	0.73
Baseline + MLR	0.787 (0.741–0.834)	+0.000	0.82	0.321	0.92

*Note:* ΔAUC, change in AUC compared with the baseline model. *p*‐values for model comparison were calculated using DeLong's test.

Abbreviations: AUC, area under the receiver operating characteristic curve; CI, confidence interval.

**Figure 4:**
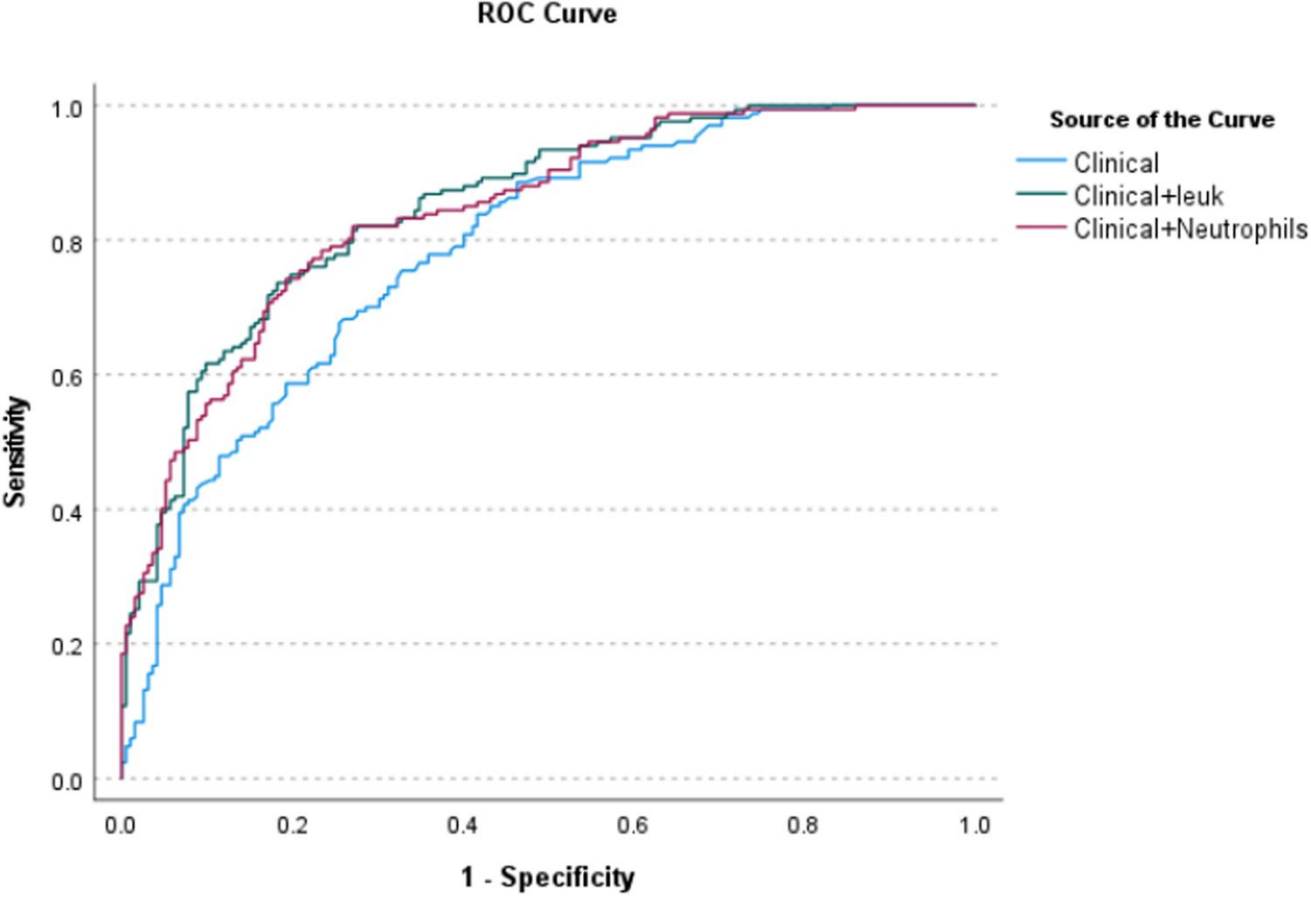
Receiver operating characteristic (ROC) curves comparing the diagnostic performance of the baseline model, the baseline plus leukocyte model, and the baseline plus neutrophil model for predicting acute coronary syndrome (ACS). The baseline model includes age, sex, smoking status, diabetes, hypertension, family history of heart disease, and previous coronary stent implantation, whereas the combined models incorporate leukocyte count or neutrophil count in addition to the baseline variables. The x‐axis represents 1 − specificity, and the y‐axis represents sensitivity. Diagnostic performance was assessed by the area under the ROC curve (AUC), with higher AUC values indicating better discriminative ability. The corresponding AUC values and their 95% confidence intervals are presented in Table 6.

## 4 Discussion

Our study demonstrated that leukocyte count, neutrophil count, monocyte count, MPV, and NLR were independently associated with ACS. The diagnostic performance of these variables, in descending order, was neutrophil count, leukocyte count, NLR, MPV, and monocyte count. Among these markers, only leukocyte count and neutrophil count demonstrated clinically meaningful diagnostic utility. Although these indices were significantly associated with ACS, they should be considered adjunctive rather than stand‐alone diagnostic tools.

The significant association between inflammatory CBC‐derived indices and ACS observed in the present study supports the hypothesis that systemic inflammation contributes to plaque destabilization and myocardial ischemia. In line with this concept, previous observational studies and systematic reviews have investigated the role of inflammatory indices such as NLR and absolute neutrophil and leukocyte count [26, 27, 28, 29, 30], with findings consistent with the present study. Collectively, these findings support the use of inflammatory CBC‐derived indices as adjunctive markers for the early evaluation of ACS. Conversely, Gunes et al. reported that although NLR was significantly correlated with troponin levels, it did not demonstrate sufficient diagnostic accuracy for identifying NSTEMI [23]. This discrepancy may be explained by differences in study population and diagnostic criteria, as Gunes et al. focused on NSTEMI, whereas the present study evaluated the diagnostic value of CBC‐derived markers across the broader spectrum of ACS using comprehensive clinical assessment in addition to cardiac biomarkers.

MPV was significantly associated with ACS in the present study, whereas platelet count showed no diagnostic value. This finding is biologically plausible because elevated MPV has been shown to reflect increased platelet activation and accelerated platelet turnover following atherosclerotic plaque rupture, with enhanced bone marrow thrombopoiesis compensating for platelet consumption by releasing larger, more reactive platelets into the circulation during thrombus formation [31, 32, 33]. In contrast, Gunes et al. did not identify a significant association between MPV and ACS [23]. The systematic review by Haque et al. reported an increased absolute platelet count in patients with ACS and proposed that this finding represents a component of the inflammatory response involved in the development of ACS [29]. In contrast, platelet count was not associated with ACS in the present study, suggesting that platelet activity rather than platelet quantity may be more relevant during the acute phase of ACS.

An elevated NLR reflects the combined effects of neutrophilia and lymphopenia, while an increased leukocyte count and the subsequent rise in neutrophil count during the early phase of ACS further support the role of these inflammatory markers in disease pathophysiology [34, 35]. Similarly, larger platelets exhibit greater metabolic and enzymatic activity and release higher levels of procoagulant mediators, providing a biological explanation for the observed association between MPV and ACS. This finding further supports the concept that platelet activity, rather than platelet count, is more closely related to the acute thrombotic process [29, 36, 37].

Although CBC‐derived biomarkers cannot replace cardiac troponin, the present findings suggest that leukocyte count and neutrophil count may serve as inexpensive and readily available adjunctive markers for the early evaluation of suspected ACS, particularly in resource‐limited settings.

Recent cardiovascular research has increasingly focused on composite inflammatory and immune‐nutritional biomarkers that integrate multiple biological pathways into a single index. In addition to inflammatory indices such as the systemic immune‐inflammation index (SII) and systemic inflammation response index (SIRI), nutritional‐immune indices such as the prognostic nutritional index (PNI) and controlling nutritional status (CONUT) score have gained attention. These indices combine hematological parameters with nutritional markers and have been associated with mortality and major adverse cardiovascular events in patients with coronary artery disease and other cardiovascular conditions. Nevertheless, simple CBC‐derived markers remain attractive because of their low cost, wide availability, and ease of interpretation, and future studies comparing their diagnostic performance with composite inflammatory and immune‐nutritional biomarkers may further clarify their incremental clinical value [38, 39, 40, 41].

CBC‐derived indices have also been investigated for prognostic stratification in patients with ACS. For example, the hemoglobin‐to‐red cell distribution width ratio was associated with 30‐day mortality and major adverse cardiovascular events (MACE) in emergency department patients with ACS [42]. Such findings support the potential clinical relevance of hematologic indices in ACS; however, prognostic associations should not be interpreted as evidence of diagnostic accuracy at presentation. Therefore, the diagnostic utility of CBC‐derived markers should be evaluated separately from their prognostic value.

Beyond their diagnostic utility in ACS, CBC‐derived inflammatory indices may also have broader prognostic implications in patients with cardiovascular disease. Previous studies have demonstrated associations between systemic inflammatory indices and adverse clinical outcomes, including contrast‐induced nephropathy or acute kidney injury following percutaneous coronary intervention, as well as in‐stent restenosis. These findings suggest that inflammatory and hematologic indices may provide clinically relevant information beyond the initial diagnosis of ACS and may help identify patients at higher risk of subsequent cardiovascular or renal complications. However, the present study focused primarily on the diagnostic value of CBC‐derived parameters in ACS, and these long‐term prognostic outcomes were not evaluated. Future prospective studies are therefore warranted to investigate the prognostic role of these indices and their potential clinical utility in risk stratification [43, 44, 45].

Despite the growing body of evidence, including the findings of the present study, CBC‐derived indices have not yet been incorporated into standard diagnostic algorithms for ACS. Although these markers have demonstrated significant associations with ACS, the current evidence remains insufficient to support their routine clinical use. Therefore, further large‐scale, well‐designed studies are warranted to validate their diagnostic performance and clinical utility.

### 4.1 Limitations & Strengths

This study has several limitations. First, its retrospective, single‐center design may have introduced selection bias and limits the external generalizability of the findings. Second, angiographic data were unavailable because the study center lacked angiography facilities. Third, although multivariable logistic regression was performed, residual confounding from unmeasured factors, including subclinical inflammatory conditions, comorbidities, and medication use, cannot be excluded. Furthermore, ACS diagnosis was established by the treating cardiologist according to guideline‐based criteria without independent blinded adjudication, which may have introduced diagnostic variability. Finally, internal and external validation of the diagnostic performance of these CBC‐derived indices was not performed, and their relatively modest diagnostic performance suggests that these markers should be considered complementary rather than stand‐alone diagnostic tools. As SII, SIRI, PNI, and CONUT were not evaluated in this study, future studies should consider investigating these indices.

## 5 Conclusion

This study demonstrated that several CBC‐derived inflammatory indices, including leukocyte count, neutrophil count, NLR, MPV, and monocyte count, were significantly associated with ACS. However, their overall diagnostic performance was limited, and only leukocyte count and neutrophil count demonstrated clinically meaningful diagnostic utility. Therefore, these biomarkers should be considered adjunctive rather than stand‐alone diagnostic tools for the evaluation of patients with suspected ACS. Given their low cost, wide availability, and rapid turnaround time, they may provide complementary information, particularly in resource‐limited settings where advanced cardiac biomarkers are not readily accessible. Further prospective, multicenter studies with external validation are required before these indices can be recommended for routine clinical use.

## Author Contributions

**Mohammad Ghorbani:** conceptualization, investigation, validation, methodology, supervision, writing – review and editing, writing – original draft. **Mostafa Shakeri:** investigation, writing – original draft, writing – review and editing, methodology, project administration, data curation, software, conceptualization, validation, visualization. **Elahe Rahimi:** methodology, validation, software, formal analysis, supervision, writing – review and editing, writing – original draft, investigation. **Ebrahim Salavati:** supervision, validation. **Raheleh Derafshi:** supervision, validation. **Mohamad Esmail Gheydari:** supervision, validation. **Amirhossein Gholizadeh Behabadi:** investigation.

## Funding

The authors have nothing to report.

## Ethics Statement

Ethical approval was obtained from the Ethics Committee of Gonabad University of Medical Sciences under approval code IR.GMU.REC.1402.168. Written informed consent was obtained from all volunteers who participated in this study. Written informed consent was obtained from the parents or legal guardians of the adolescent participants, and assent was obtained from the participants when appropriate. This study was also conducted in accordance with the relevant guidelines and regulations, including the Declaration of Helsinki.

## Conflicts of Interest

The authors declare no conflicts of interest.

## Author Accountability Statement

All authors have read and approved the final version of the manuscript. Mostafa shakeri had full access to all of the data in this study and takes complete responsibility for the integrity of the data and the accuracy of the data analysis.

## Transparency Statement

The lead author Mostafa Shakeri affirms that this manuscript is an honest, accurate and transparent account of the study being reported; that no important aspects of the study have been omitted; and that any discrepancies from the study as planned (and, if relevant, registered) have been explained.

## Data Availability

The data supporting this study's findings are available on request from the corresponding author. The data are not publicly available due to privacy or ethical restrictions.
